# Malic Enzyme 1 Limits Acetaminophen-Induced Liver Injury by Sustaining Redox and Bioenergetic Homeostasis

**DOI:** 10.3390/metabo16060423

**Published:** 2026-06-16

**Authors:** Chang Guo, Zizhi Tang

**Affiliations:** 1 Institute of Basic Medical Sciences & School of Basic Medicine, Chinese Academy of Medical Sciences & Peking Union Medical College, Beijing 100730, China; 2 College of Life Science, Sichuan University, Chengdu 610065, China

**Keywords:** malic enzyme 1, acetaminophen, APAP-induced liver injury, NADPH, redox homeostasis, mitochondrial respiration, autophagy, ER stress, AMPK/mTOR signaling, hepatoprotection

## Abstract

**Background**: Acetaminophen (APAP) overdose remains a major cause of acute liver injury. Although N-acetylcysteine (NAC) is the clinically established antidote for APAP toxicity, its efficacy is greatest when administered early, and additional therapeutic strategies are still needed for patients with delayed presentation or progressive injury. Because APAP hepatotoxicity involves coupled disturbances in redox control, mitochondrial performance, and cellular metabolism, metabolic enzymes that sustain NADPH availability may critically influence disease severity. Malic enzyme 1 (ME1), a cytosolic NADPH-generating enzyme, has not been functionally defined in this context. **Methods**: To determine the contribution of ME1 to APAP-induced liver injury (AILI), we used hepatocyte-specific ME1 knockout mice, hepatic overexpression and reconstitution approaches, primary mouse hepatocytes, and an enzymatically inactive ME1 mutant. Liver injury and associated changes in oxidative stress, mitochondrial function, energy metabolism, autophagic flux, and endoplasmic reticulum (ER) stress were evaluated using biochemical, histological, molecular, and ultrastructural analyses, together with pharmacological interventions. **Results**: Genetic loss of ME1 did not substantially alter early APAP metabolic activation-related indices, including APAP-protein adduct formation, but markedly increased hepatocellular metabolic vulnerability after APAP challenge. This phenotype was characterized by enhanced lipid peroxidation, impaired mitochondrial polarization, reduced ATP availability, defective autophagic flux, and amplified ER stress, leading to more severe liver damage. In contrast, ME1 overexpression or reconstitution promoted a more adaptive metabolic response and limited tissue injury. These effects depended largely on ME1 catalytic activity, as protection was markedly weakened with the mutant enzyme. Pharmacological analyses further supported the involvement of AMPK/mTOR-associated autophagy regulation and ER stress adaptation in the downstream actions of ME1. Malic acid also partially attenuated APAP-induced hepatotoxicity in vivo and in vitro. **Conclusions**: ME1 functions as an endogenous metabolic factor that influences the outcome of APAP-induced liver injury. Its catalytic activity supports hepatocyte survival primarily by preserving reductive capacity, bioenergetic balance, and adaptive stress responses, rather than by altering APAP metabolic activation.

## 1. Introduction

Mammalian malic enzymes comprise three isoforms with distinct subcellular localization and cofactor preference. ME1 is a cytosolic NADP+-dependent malic enzyme; ME2 is primarily mitochondrial and can use NAD+ and NADP+ as cofactors, whereas ME3 is a mitochondrial NADP+-dependent isoform. ME1 catalyzes the oxidative decarboxylation of malate to pyruvate with concomitant NADPH generation and is classified as malate dehydrogenase (oxaloacetate-decarboxylating, NADP+) (EC 1.1.1.40). Because NADPH is required for glutathione- and thioredoxin-dependent antioxidant systems, ME1 may contribute to cytosolic NADPH supply and participate in redox-sensitive stress adaptation [1,2]. In recent years, ME1 has been implicated not only in metabolic reprogramming but also in stress adaptation and cell survival under pathological conditions [1]. Notably, ME1 has been reported to limit ferroptosis-associated liver damage in hepatic ischemia/reperfusion injury. These observations raise the possibility that ME1 may also contribute to hepatocyte protection under acute injury conditions [3].

Excessive acetaminophen (APAP) intake continues to serve as a key etiological driver of acute hepatic injury and acute liver failure. N-acetylcysteine (NAC) remains the most effective and clinically established antidote for APAP toxicity, primarily by supporting glutathione restoration; however, its benefit is greatest when administered early, and therapeutic challenges remain in patients who present late after overdose or develop progressive injury [4,5,6]. A key early step in APAP-induced hepatotoxicity is the cytochrome P450-dependent production of the reactive metabolite N-acetyl-p-benzoquinone imine (NAPQI). Once generated, NAPQI depletes glutathione and conjugates to cellular proteins, thereby inducing mitochondrial oxidative stress, energetic disturbance, and ultimately necrotic cell death [6,7,8,9]. For this reason, mitochondrial dysfunction, mitochondrial oxidant stress, and disruption of intracellular redox balance are central early features of AILI. They are likely to shape later stress responses in injured hepatocytes [7,8].

Among the downstream events implicated in AILI, endoplasmic reticulum (ER) stress and autophagy have received particular attention. ER stress is increasingly recognized as a contributor to APAP-induced liver injury. In contrast, autophagy is generally regarded as an adaptive response that facilitates the clearance of damaged organelles and toxic protein adducts [10,11,12]. In parallel, AMPK/mTOR-associated signaling has emerged as an important regulatory node linking metabolic stress to autophagic responses [10,13,14,15,16,17]. Although previous studies have found that modulation of ER stress or autophagy may influence the severity of APAP hepatotoxicity, these investigations have largely focused on individual downstream pathways. However, the upstream metabolic factors that coordinate redox homeostasis with autophagic and ER stress responses in APAP-induced liver injury (AILI) remain incompletely defined [18,19].

Given that ME1 can contribute to cytosolic NADPH production and that redox imbalance is an important feature of AILI, we postulated that hepatocyte-derived ME1 may influence APAP-induced liver injury by modulating downstream redox-sensitive stress adaptation, rather than acting as the predominant source of hepatic NADPH. To evaluate the role of ME1 in AILI, we used ME1-deficiency and overexpression models and a catalytically inactive ME1 mutant. We next examined whether the protective effect of ME1 was coupled with perturbations in redox balance and downstream cellular stress signaling cascades, encompassing AMPK/mTOR-linked signaling, autophagic turnover, and endoplasmic reticulum (ER) stress [10].

## 2. Materials and Methods

### 2.1. Animal Experiments

C57BL/6J mice were acquired from the Experimental Animal Center of the Chinese Academy of Medical Sciences. Alb-Cre mice were obtained from Shulaibao Biotechnology Co., Ltd. (Wuhan, China). and were crossed with ME1 fl/fl mice to generate ME1ΔHC mice with hepatocyte-specific ME1 deletion. Genotypes were determined by PCR analysis of genomic DNA obtained from tail biopsies. ME1fl/fl littermates lacking Alb-Cre were used as WT controls, whereas Alb-Cre; ME1fl/fl mice were used as hepatocyte-specific ME1 knockout mice (ME1ΔHC). Only mice with confirmed genotypes and without signs of illness, abnormal body weight, or poor general condition were included in the experiments. Healthy male mice (8 weeks old) were used. All mice were kept in an SPF facility at a constant 25 °C, with free access to standard chow and water. The animal experiments performed in this study were granted ethical approval by the Shouzheng Hongyao Animal Ethics Committee (No. SZHY Dong (Fu) 2023100101). All protocols conducted in the present study were performed in full compliance with the animal care and use guidelines and the requirements set forth in the national standard (GB/T 35892-2018) [20].

Acetaminophen (APAP, catalog number A7302, Sigma-Aldrich) was solubilized in 0.9% (*w*/*v*) sodium chloride (saline) preheated to 55 °C, then allowed to equilibrate to 37 °C prior to administration. After overnight fasting, mice received an injection of APAP at either 250 or 500 mg/kg, depending on the experimental design. Unless otherwise indicated, serum and mouse tissues were collected 24 h after APAP administration. For survival experiments, mice were challenged with a lethal dose of APAP (i.p., 500 mg/kg). To suppress endoplasmic reticulum (ER) stress, tauroursodeoxycholic acid (TUDCA, catalog number M5158, AbMole) was delivered at a dose of 250 mg/kg. For pharmacological intervention studies with malic acid, mice were administered malic acid (catalog number PHR1273-1G, Sigma-Aldrich) by intraperitoneal injection at 300 mg/kg 1 h prior to the APAP challenge.

A total of 100 mice were included under the approved animal protocol. Animals used for tissue collection were shared across compatible biochemical, histological, molecular, and ultrastructural analyses whenever possible, and group sizes for each experimental endpoint are provided in the corresponding figure legends. All animal experiments were carried out in a randomized, blinded fashion to prevent subjective observer bias.

### 2.2. Adenoviral Constructs and In Vivo Delivery

To prepare the adenoviral constructs, the mouse ME1 coding sequence was subcloned into the pAd-CMV vector, followed by independent adenovirus packaging. For in vivo adenoviral delivery, mice received 10^9^ pfu of either the control adenovirus (ADV-RAM) or the ME1-overexpression adenovirus (ADV-ME1) by tail-vein injection 5 days prior to APAP treatment.

To generate a catalytically inactive form of ME1, lysine 269 was substituted with alanine by site-directed mutagenesis. The K269A mutant was then inserted into the same adenoviral vector and packaged for rescue studies.

### 2.3. Assessment of Serum ALT and AST Levels

Serum ALT (BC1555, Solarbio, Beijing, China) and AST (BC1565, Solarbio, Beijing, China) levels were determined by commercial kits following the manufacturers’ instructions.

### 2.4. Immunoblotting

RIPA lysis buffer (20101ES60, Yeasen) with protease inhibitors (PI) was used for total protein extraction. Samples were incubated for 40 min on ice, vortexed three times, and then centrifuged at 4 °C at 12,000× *g* for 5 min. Total protein concentrations were determined by the BCA assay kit (Yeasen, 20201ES76). Equal amounts of protein were separated by SDS-PAGE, and the proteins were transferred onto a methanol-activated PVDF Membrane (Roche, 03010040001). Subsequently, the membranes were blocked using 5% BSA and incubated overnight with the corresponding primary antibodies in TBST buffer; antibody details are summarized in Table 1. Based on the molecular mass of the different target proteins, membranes were processed differently; in some cases, they were cut before antibody incubation. When proteins of similar size were examined, or when total and phosphorylated forms were analyzed, membranes were stripped and reprobed when needed. The intensities of protein bands were quantified using ImageJ (ImageJ 1.53) software. Non-phosphorylated proteins were normalized to GAPDH unless otherwise indicated. Phosphorylated proteins were normalized to their corresponding total proteins, including p-PERK/PERK, p-eIF2α/eIF2α, p-AMPK/AMPK, p-S6/S6, and p-ULK1/ULK1. APAP-protein adduct signals were normalized to GAPDH.

### 2.5. Quantitative Real-Time PCR

Total RNA was from primary cells and liver tissue specimens with a commercial extraction kit (ESScience, catalog no. RN001-50Rxns). Subsequently, 3 μg of purified RNA was reverse-transcribed to cDNA using a RT-PCR kit (catalog no. R211-01, Vazyme). mRNA levels were quantified by qRT-PCR using qRT-PCR Master Mix (catalog no. 11199ES03, Yeasen), and relative expression was calculated using the 2^−ΔΔCt^ method. Gene expression levels were normalized against the housekeeping gene GAPDH, and each assay was conducted in technical triplicate with three independent biological replicates to ensure statistical reliability. Primers are shown in Table 2.

### 2.6. Histology and Immunostaining

For immunofluorescence (IF) assays, cells cultured on glass coverslips were first fixed in 4% (*w*/*v*) paraformaldehyde for 15 min, followed by using 1% Triton X-100 dissolved in PBS, with the concentration selected based on experimental requirements. Tissue sections and cell samples mounted on slides were blocked in TBST buffer containing 5% BSA and incubated overnight at 4 °C with primary antibodies specific for ME1 (catalog no. 16619-1-AP, Proteintech, Wuhan, China) and CHOP (product no. 15204-1-AP, Proteintech, Wuhan, China). Sections were incubated with the ESScience SGAR488 secondary antibody at room temperature for the recommended duration to ensure specific signal detection. Nuclei were counterstained with DAPI to visualize chromatin architecture. Fluorescence images were collected using Leica Stellaris 5, and all imaging parameters were kept consistent across experimental groups.

Hematoxylin and eosin (H&E) staining and Immunohistochemical (IHC) staining were performed by Servicebio Technology Co., Ltd. (Wuhan, China). For quantitative analysis of IHC staining, digital images were acquired under identical imaging conditions. CHOP- or ME1-positive areas were quantified using ImageJ by applying the same color threshold across all groups. The percentage of positive area was calculated as the positively stained area divided by the total tissue area within each field. Multiple randomly selected fields from each section were analyzed by investigators blinded to group allocation.

### 2.7. Isolation and Treatment of Primary Hepatocytes

Mouse primary hepatocytes were isolated from 8-week-old mice by a two-step collagenase perfusion method. Briefly, mice were anesthetized, and the liver was perfused in situ through the portal vein with prewarmed calcium-free perfusion buffer to remove blood, followed by digestion with collagenase IV-containing buffer. After digestion, the liver was excised, gently disrupted, and the cell suspension was filtered through a cell strainer. Hepatocytes were collected by low-speed centrifugation, washed, and seeded in Williams’ Medium E containing 10% FBS. Cells were allowed to attach for 6 h and were then maintained in a humidified incubator at 37 °C with 5% CO_2_ for subsequent experiments. Cell preparations with poor attachment or obvious contamination were excluded. Each in vitro experiment was repeated using hepatocytes from at least three independent isolations, as indicated in the figure legends.

For in vitro injury experiments, hepatocytes were treated with APAP (10 mM, dissolved in DMSO) for the indicated times depending on the experimental endpoint. For rescue experiments, ME1-deficient hepatocytes were infected with control adenovirus, wild-type ME1 adenovirus, or K269A mutant adenovirus after attachment, and APAP treatment was initiated after adequate transgene expression.

For pharmacological intervention studies, cells were subjected to targeted treatment in line with the experimental design: N-acetylcysteine (NAC, 5 mM) was administered for a 1-h incubation, 5-aminoimidazole-4-carboxamide ribonucleotide (AICAR, 0.5 mM, catalog no. S1515, Beyotime) for 30 min, and Compound C (10 μM, product no. BLM-275, Selleck) for a 30-min pretreatment. Rapamycin (100 nM) and 3-methyladenine (3-MA, 5 mM) were utilized in the corresponding experimental groups per the study protocol. For autophagic flux assessment, primary hepatocytes were treated with APAP as indicated and then exposed to bafilomycin A1 (BafA1, 100 nM) for 0, 2, 4, or 6 h before cell harvesting. LC3-II accumulation after BafA1 treatment was used to evaluate autophagic flux. ΔLC3-II was calculated as the increase in LC3-II abundance after BafA1 treatment relative to APAP-treated cells without BafA1 within the same genotype. To further assess lysosomal proteolytic maturation, pro-CTSD and mature CTSD protein levels were examined by immunoblotting, and the mature CTSD/pro-CTSD ratio was quantified. Functionally, AICAR and Compound C were employed to modulate AMPK signaling, rapamycin and 3-MA were utilized to regulate autophagy-associated responses, and bafilomycin A_1_ was applied for autophagic flux analysis.

### 2.8. siRNA-Mediated AMPKα Knockdown and Adenoviral ME1 Reconstitution in Primary Hepatocytes

For AMPKα knockdown experiments, primary mouse hepatocytes were isolated and seeded as described above. After cell attachment, hepatocytes were transfected with mouse AMPKα1/2 siRNA (sc-45313, Santa Cruz Biotechnology, Dallas, TX, USA) or a non-targeting control siRNA using Lipofectamine 3000 according to the manufacturer’s instructions. The final siRNA concentration was 10 nM. After 48 h of siRNA transfection, hepatocytes were transduced with adenoviruses encoding wild-type ME1, the catalytically inactive K269A-ME1 mutant, or the corresponding control vector as indicated. After an additional 48 h of adenoviral expression, cells were exposed to APAP for the indicated duration and then harvested for immunoblot analysis. AMPKα knockdown efficiency was verified by immunoblotting, and downstream AMPK/mTOR-autophagy-associated signaling was evaluated by detecting p-S6, S6, p-ULK1 Ser555, p-ULK1 Ser757, ULK1, CHOP, p62, ME1, AMPKα, and GAPDH.

### 2.9. Cycloheximide Chase and Subcellular Fractionation

To evaluate the stability of WT-ME1 and K269A-ME1 proteins, primary hepatocytes expressing WT-ME1 or K269A-ME1 were treated with cycloheximide (CHX, 50 μg/mL) for 0, 4, or 8 h before harvesting. Total protein was extracted as described above, and ME1 protein levels were examined by immunoblotting. Densitometric analysis was performed using ImageJ, and ME1 levels were normalized to GAPDH and expressed relative to the 0 h time point within each group. For subcellular fractionation, cytosolic and mitochondrial fractions were isolated from hepatocytes expressing WT-ME1 or K269A-ME1 using a Mitochondria Isolation Kit for Cultured Cells (89874, Thermo Scientific, Waltham, MA, USA) according to the manufacturer’s protocol. Whole-cell lysate, cytosolic, and mitochondrial fractions were subjected to immunoblotting to examine ME1 distribution. GAPDH and TOM20 were used as cytosolic and mitochondrial markers, respectively, to assess fraction enrichment.

### 2.10. GSH and GSSG

We used a commercial kit (BC1175, Solarbio, Beijing, China) to detect the level of GSH and GSSG.

### 2.11. MDA

Cellular MDA levels were determined with an MDA detection kit (S0131M, Beyotime, Shanghai, China) following the manufacturer’s recommended protocol.

### 2.12. Measurement of Mitochondrial ROS and GSR Activity

Mitochondrial ROS levels were measured in primary hepatocytes after the indicated treatments using MitoSOX Red mitochondrial superoxide indicator (S0033S, Beyotime, Shanghai, China) according to the manufacturer’s instructions. Fluorescence intensity was measured under identical acquisition settings and normalized to the indicated control group.

Glutathione reductase (GSR) activity was measured using a commercial GSR activity assay kit (GRSA, Sigma-Aldrich, St. Louis, MO, USA) according to the manufacturer’s protocol. Enzyme activity was normalized to total protein content and expressed as specific activity (nmol NADPH/min/mg protein).

### 2.13. Seahorse Extracellular Flux Analysis

Mitochondrial respiration was assessed using a Seahorse XF extracellular flux analyzer (XFe24, Agilent Technologies, Santa Clara, CA, USA) with the Seahorse XF Cell Mito Stress Test Kit according to the manufacturer’s instructions. Primary hepatocytes were seeded in Seahorse XF cell culture microplates at a density of 1 × 10^4^ cells/well and subjected to the indicated treatments. Before the assay, the culture medium was replaced with Seahorse XF assay medium supplemented with 10 mM glucose, 1 mM pyruvate, and 2 mM L-glutamine, and cells were incubated at 37 °C in a non-CO_2_ incubator for 1 h. No exogenous L-malate was added, and the assay was performed in intact hepatocytes under mixed substrate availability rather than as a malate-specific respiration assay. OCR was measured under basal conditions and after sequential injection of oligomycin, FCCP, and rotenone/antimycin A. The final well concentrations were 1.5 μM oligomycin, 1.0 μM FCCP, and 0.5 μM rotenone/antimycin A; the FCCP concentration was selected based on preliminary optimization. Basal respiration, ATP-linked respiration, maximal respiration, and spare respiratory capacity were calculated according to the standard Seahorse Cell Mito Stress Test workflow and normalized to cell number.

### 2.14. CCK-8 Assay

A commercial CCK-8 assay kit was used to evaluate the viability of hepatocytes, according to the manufacturer’s protocol.

### 2.15. Determination of NAPQI Levels and APAP-Protein Adduct Detection

NAPQI concentrations were quantified using an enzyme-linked immunosorbent assay kit (CB12021-Mu, COIBO BIO, Shanghai, China) according to the manufacturer’s protocol. Standard curves were generated for each plate, and all samples were tested in triplicate wells. Measurements were accepted only when the standard curve met the manufacturer’s quality-control criteria and replicate variation was within the acceptable range specified by the kit protocol. APAP-protein adducts were examined by immunoblotting. Liver tissues were collected at 0, 2, and 4 h after APAP administration, and total protein was extracted as described above. Equal amounts of protein were separated by SDS-PAGE and transferred to PVDF membranes. APAP-protein adducts were detected using an anti-APAP-protein adduct antibody (Bio-Rad, 0016-0104 Hercules, CA, USA) according to the manufacturer’s instructions. GAPDH was used as the loading control, and band intensities were quantified using ImageJ.

### 2.16. ME1 Enzymatic Activity Assay

The activity of malic enzyme 1 (ME1) was quantified with an assay kit (Solarbio, BC1125, Beijing, China) by monitoring the generation of NADPH.

### 2.17. NADPH/NADP Measurement

Intracellular NADPH and NADP+ levels were measured using a commercial NADP+/NADPH assay kit (Beyotime, S0179, Shanghai, China) according to the manufacturer’s instructions. Briefly, primary hepatocytes were collected after the indicated treatments and lysed in the extraction buffer provided with the kit. Total NADP(H) and NADPH were measured according to the kit protocol. NADP+ levels were calculated by subtracting NADPH from total NADP(H), and the NADPH/NADP+ ratio was calculated for each sample. Values were normalized to protein concentration as indicated.

### 2.18. JC-1 Staining and Energy Metabolite Measurements

Mitochondrial membrane potential (MMP) was analyzed via JC-1 staining utilizing a commercially available assay kit (C2006, Beyotime, Shanghai, China), following the detailed instructions provided by the manufacturer. Intracellular ATP and ADP concentrations were determined using separate commercial kits from Beyotime (China, catalog no. S0026), and the resultant ATP/ADP ratio was subsequently calculated for each sample.

### 2.19. Transmission Electron Microscopy Experiment

For transmission electron microscopy, liver tissues were fixed in 2.5% glutaraldehyde, post-fixed in 1% osmium tetroxide, dehydrated through a graded ethanol series, and embedded in epoxy resin. Ultrathin sections were stained with uranyl acetate and lead citrate and examined under a transmission electron microscope (JEM-1400, JEOL, Tokyo, Japan) at 80 kV. Representative images were selected from at least three mice per group.

### 2.20. Statistical Analysis

Quantitative data are reported as the mean ± standard deviation (SD) across all analyses. Statistical evaluations were performed using a methodology tailored to the experimental design: unpaired Student’s *t*-tests were applied for direct comparisons between two independent groups. One-way analysis of variance (ANOVA) followed by Tukey’s post hoc multiple comparisons test was used to correct for multiple comparisons among three or more groups. Kaplan–Meier analysis was conducted to generate survival curves, and statistical significance was defined as a *p*-value < 0.05. Exact *p* values are provided in Supplementary Table S1 where practical; otherwise, significance levels are indicated using threshold notation in the figures. All sample sizes (n values) are specified in the corresponding figure legends.

## 3. Results

### 3.1. Hepatocyte-Specific Loss of ME1 Aggravates, Whereas Hepatic Overexpression or Reconstitution of ME1 Attenuates, APAP-Induced Liver Injury In Vivo

**Figure 1:**
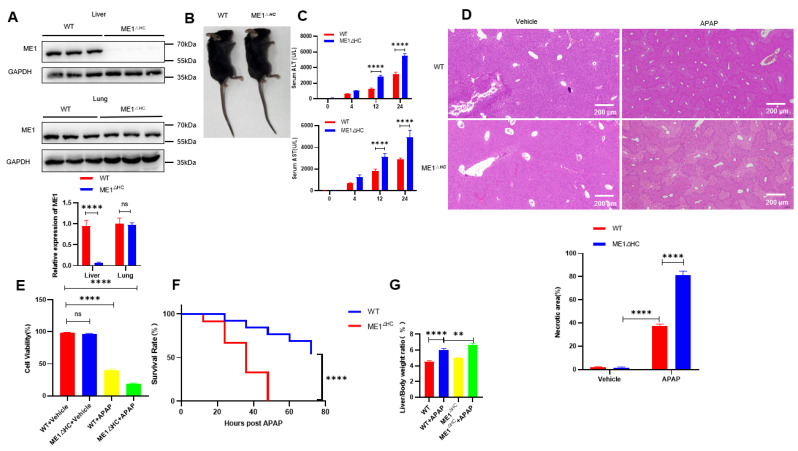
Hepatocyte-specific deletion of ME1 exacerbates APAP-induced acute liver injury in vivo. WT and ME1ΔHC mice were treated with APAP as indicated. (**A**) Immunoblot analysis of ME1 expression in liver and extrahepatic tissues to confirm hepatocyte-specific deletion of ME1 in ME1ΔHC mice, together with densitometric quantification (n = 6 per group). (**B**) Representative gross appearance of WT and ME1ΔHC mice under basal conditions. (**C**) Serum ALT and AST concentrations were assessed in WT and ME1ΔHC mice 24 h post APAP challenge (n = 5 per group). (**D**) Hepatic necrosis was visualized by H&E staining of mouse liver tissue sections (scale bar: 200 μm), and the area of necrotic lesions was quantified across all experimental groups (n = 5 per group). (**E**) Viability of primary hepatocytes isolated from WT and ME1ΔHC mice after APAP exposure (n = 3 independent experiments). (**F**) Survival curves of WT and ME1ΔHC mice following lethal APAP challenge (n = 12 per group). (**G**) Liver/body weight ratio in WT and ME1ΔHC mice with or without APAP (n = 5 mice per group). All quantitative data are presented as the mean ± standard deviation (SD). For statistical analysis: comparisons between two independent groups were performed by unpaired two-tailed Student’s *t*-tests; multiple groups were analyzed via one-way ANOVA followed by Tukey’s post hoc test, and survival curves were evaluated with the log-rank test. ns, not significant; ** *p* < 0.01, **** *p* < 0.0001 as indicated.

To ascertain whether hepatocellular ME1 influences the severity of acetaminophen-induced liver injury, we used complementary loss-of-function and gain-of-function approaches in vivo. Immunoblotting was performed to confirm the deletion of ME1 in liver tissue, while no comparable deletion was observed in extrahepatic tissues. In addition to the lung (Figure 1A), ME1 expression in the kidney and heart was preserved in ME1ΔHC mice (Figure S1), supporting hepatocyte-restricted ME1 deletion. ME1-deficient mice appeared grossly normal under baseline conditions (Figure 1B). However, after APAP administration, these mice developed substantially more severe liver injury than their wild-type counterparts, as indicated by higher serum aminotransferase activities (Figure 1C), broader necrotic regions in liver sections (Figure 1D), reduced viability of primary hepatocytes (Figure 1E), and increased mortality following a lethal APAP challenge (Figure 1F), and a higher liver/body weight ratio after APAP exposure (Figure 1G). Together, these findings indicate that hepatocyte-derived ME1 protects against APAP-mediated hepatic injury and contributes to hepatocyte stress adaptation under toxic conditions.

**Figure 2:**
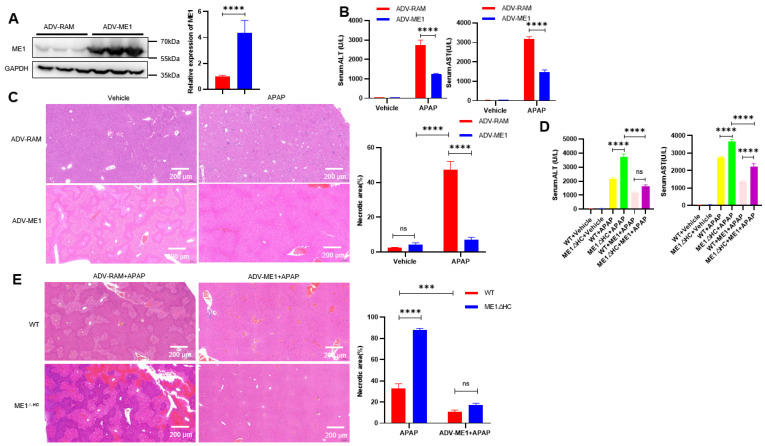
Ectopic hepatic overexpression or re-expression of ME1 protects against APAP-induced hepatic injury in mice. Five days prior to APAP administration (250 mg/kg, i.p.), WT and ME1ΔHC mice were transduced with either control adenovirus (ADV-RAM) or adenovirus encoding ME1 (ADV-ME1). (**A**) Hepatic ME1 protein levels were evaluated via immunoblotting in ADV-RAM- and ADV-ME1-treated mice, with densitometric analysis of target bands provided (n = 6 mice per group). (**B**) To explore the hepatoprotective potential conferred by ME1 overexpression, serum ALT and AST levels were measured in WT mice administered ADV-RAM or ADV-ME1 adenovirus, followed by vehicle or APAP-induced liver injury (n = 5 per group). (**C**) Representative H&E-stained liver sections from WT mice (scale bar: 200 μm) demonstrated that ME1 overexpression significantly attenuated APAP-induced hepatic necrosis; quantitative analysis of necrotic areas is shown (n = 5 per group). (**D**) Serum ALT and AST levels in WT and ME1ΔHC mice treated with vehicle or APAP, with or without ADV-ME1-mediated re-expression (n = 5 mice per group). (**E**) Representative H&E-stained liver sections from WT and ME1ΔHC mice following APAP challenge with or without ADV-ME1 administration (scale bar: 200 μm), along with quantitative analysis of necrotic areas (n = 5 per group). Data are presented as mean ± SD. Statistical comparisons were analyzed via one-way ANOVA followed by Tukey’s post hoc test. ns, not significant; *** *p* < 0.001; **** *p* < 0.0001, as indicated.

We next asked whether increasing hepatic ME1 expression would produce the opposite phenotype. In wild-type mice, adenoviral delivery of ME1 (Figure 2A) before APAP exposure markedly attenuated liver injury, which was reflected by lower ALT and AST levels and a reduction in histological necrosis (Figure 2B,C). To further strengthen this conclusion, ME1 was re-expressed in hepatocyte-specific ME1-deficient mice before APAP treatment. Restoration of ME1 in this knockout background alleviated the exaggerated liver injury phenotype compared with control-treated knockout mice (Figure 2D,E). Collectively, complementary loss- and gain-of-function experiments identify ME1 as an endogenous hepatoprotective factor during APAP-induced acute liver injury.

### 3.2. APAP Challenge Induces ME1 Expression in Hepatocytes

**Figure 3:**
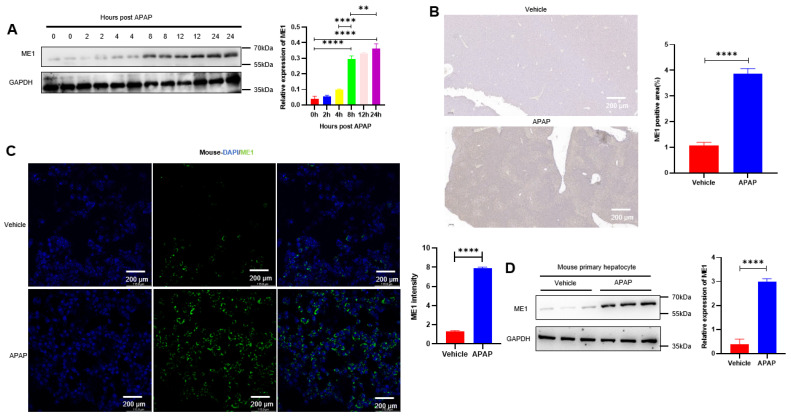
APAP challenge induces ME1 expression in the liver and primary hepatocytes. WT mice or primary hepatocytes treated with APAP as indicated. (**A**) Immunoblot analysis of hepatic ME1 protein levels in mouse livers collected at the indicated time points after APAP administration, together with densitometric quantification (n = 6 mice per time point). (**B**) Representative ME1 immunohistochemical staining in liver tissues from vehicle- or APAP-administered mice (scale bar: 200 μm), alongside quantitative analysis of ME1-positive regions (n = 5 per group). (**C**) Immunofluorescence staining of primary mouse hepatocytes showing ME1 expression after vehicle or APAP treatment (green, ME1; blue, DAPI; scale bar: 200 μm), together with quantification of ME1 fluorescence intensity (n = 3 independent experiments). (**D**) Immunoblot analysis of ME1 expression in primary hepatocytes with vehicle or APAP, together with densitometric quantification (n = 3 independent experiments). Data are presented as mean ± standard deviation (SD). All statistical analyses were performed using one-way ANOVA with Tukey’s post hoc multiple comparisons test or unpaired two-tailed Student’s *t*-test, as appropriate. ns, not significant; ** *p* < 0.01, **** *p* < 0.0001 as indicated.

In view of the protective role of ME1 revealed by the above functional experiments, we next examined whether hepatic ME1 expression is altered during APAP-induced injury. Western blot analysis showed that hepatic ME1 protein levels increased after APAP exposure (Figure 3A). Histological staining further confirmed this increase by demonstrating enhanced ME1 immunoreactivity in damaged liver tissue (Figure 3B). In parallel, immunofluorescence analysis confirmed increased ME1 expression in primary hepatocytes exposed to APAP (Figure 3C), and a similar increase was detected by immunoblotting in APAP-treated primary mouse hepatocytes in vitro (Figure 3D). These observations indicate that ME1 is inducible in hepatocytes under APAP stress, consistent with its proposed protective role in the injured liver.

### 3.3. ME1 Deficiency Does Not Substantially ALTER APAP Metabolic Activation but Aggravates Downstream Oxidative, Bioenergetic, and ER Stress Responses

**Figure 4:**
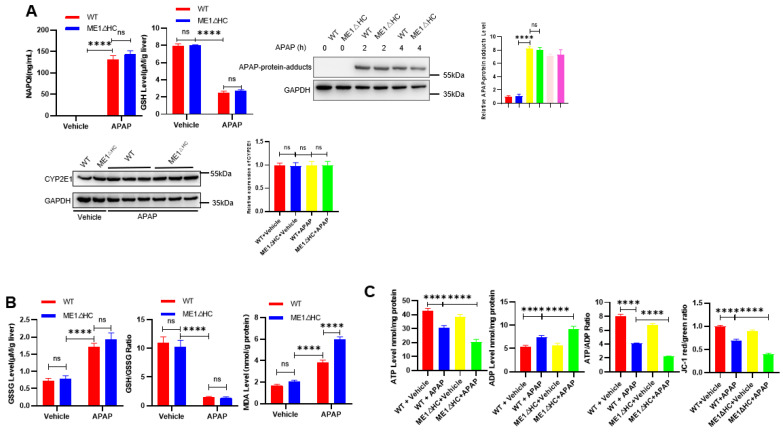
ME1 deficiency does not substantially alter APAP metabolic activation but is associated with aggravated oxidative stress and bioenergetic impairment. WT and ME1ΔHC mice were treated with vehicle or APAP as indicated. (**A**) Hepatic APAP metabolic activation-related indices were assessed, including NAPQI levels, total GSH levels, APAP-protein adduct formation, and CYP2E1 protein expression. APAP-protein adducts were detected by immunoblotting in liver tissues collected at 0, 2, and 4 h after APAP administration, with densitometric quantification normalized to GAPDH. CYP2E1 protein expression was examined by immunoblotting, with corresponding densitometric quantification (n = 5 per group). (**B**) Hepatic redox and oxidative stress-related indices, including GSSG levels, GSH/GSSG ratio, and MDA levels, were measured in the indicated groups (n = 5 per group). (**C**) Hepatic ATP levels, ADP concentrations, ATP/ADP ratio, and JC-1 red/green fluorescence ratio were quantified to evaluate bioenergetic disturbance and mitochondrial membrane potential (n = 5 per group). Data are presented as mean ± SD. Statistical comparisons were performed using one-way ANOVA followed by Tukey’s post hoc multiple comparisons test or unpaired two-tailed Student’s *t*-test where appropriate. ns, not significant; **** *p* < 0.0001, as indicated.

To elucidate the mechanism by which ME1 modulates APAP-induced hepatotoxicity, we first assessed whether hepatocyte-specific ME1 deficiency affected APAP metabolic activation. Hepatic NAPQI levels were markedly increased after APAP administration, but no significant difference was detected between WT and ME1ΔHC mice (Figure 4A). Similarly, total hepatic GSH levels were reduced after APAP challenge, whereas the extent of GSH depletion was comparable between the two genotypes (Figure 4A). To more directly evaluate APAP metabolic activation, we further examined APAP-protein adduct formation at early time points after APAP administration. APAP-protein adducts were barely detectable under basal conditions but were markedly induced at 2 and 4 h after APAP challenge. Importantly, APAP-protein adduct abundance did not differ significantly between WT and ME1ΔHC mice at either time point (Figure 4A). In addition, CYP2E1 protein expression was comparable between WT and ME1ΔHC livers under vehicle- and APAP-treated conditions (Figure 4A). Together, these findings indicate that ME1 deficiency does not substantially alter the early APAP metabolic activation-related readouts examined under our experimental conditions.

Although APAP metabolic activation-related readouts were largely comparable between genotypes, ME1 deficiency was associated with more pronounced redox and oxidative stress-related alterations after APAP challenge. APAP treatment increased hepatic GSSG levels and reduced the GSH/GSSG ratio in both WT and ME1ΔHC mice. In contrast, the differences between APAP-treated WT and ME1ΔHC mice were not statistically significant for these glutathione-related indices (Figure 4B). By contrast, MDA levels were significantly higher in ME1ΔHC livers than in WT livers after APAP administration, indicating enhanced lipid peroxidation in the absence of ME1 (Figure 4B). Moreover, ME1-deficient livers exhibited more severe bioenergetic impairment, as shown by reduced ATP levels, increased ADP levels, a lower ATP/ADP ratio, and a decreased JC-1 red/green fluorescence ratio after APAP challenge (Figure 4C). These results suggest that ME1 deficiency mainly aggravates downstream oxidative and mitochondrial stress responses rather than enhancing the initial metabolic activation of APAP.

**Figure 5:**
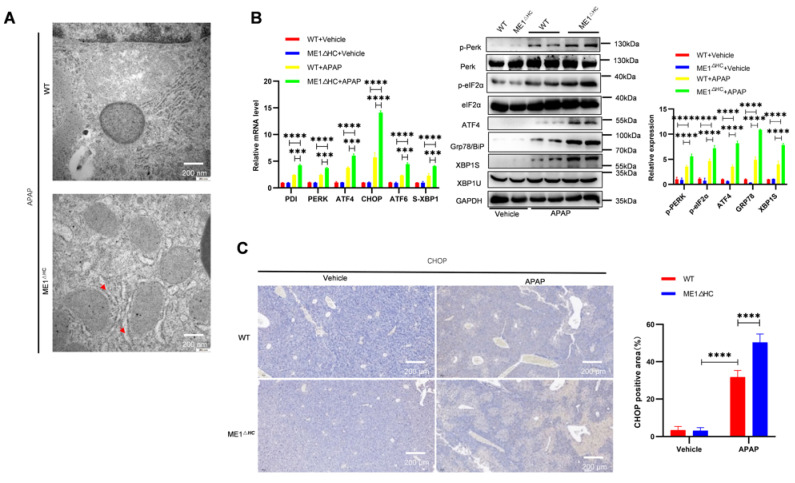
ME1 deficiency aggravates ER stress during APAP-induced liver injury. WT and ME1ΔHC mice were treated with vehicle or APAP as indicated. (**A**) Representative transmission electron microscopy images showing endoplasmic reticulum ultrastructural changes in APAP-treated WT and ME1ΔHC liver tissues. Red arrowheads indicate swollen or dilated ER structures. Scale bar: 200 nm (n = 3 per group). (**B**) qRT-PCR analysis of ER stress-related genes, including PDI, PERK, ATF4, CHOP, ATF6, and sXBP1, and immunoblot analysis of ER stress-associated proteins, including p-PERK, PERK, p-eIF2α, eIF2α, ATF4, GRP78/BiP, XBP1s, and XBP1u, with corresponding densitometric quantification. Phosphorylated proteins were normalized to their corresponding total proteins, and non-phosphorylated proteins were normalized to GAPDH (n = 5 per group). (**C**) Representative CHOP immunohistochemical staining in liver sections from WT and ME1ΔHC mice treated with vehicle or APAP, together with quantification of CHOP-positive area. Scale bar: 200 μm (n = 5 per group). Data are presented as mean ± SD; Statistical comparisons were performed using one-way ANOVA followed by Tukey’s post hoc multiple comparisons test. ns, not significant; *** *p* < 0.001; **** *p* < 0.0001, as indicated.

Because mitochondrial dysfunction and redox disturbance are closely linked to ER stress during APAP-induced liver injury, we next examined ER stress-associated changes. Transmission electron microscopy revealed more pronounced ER swelling in APAP-treated ME1ΔHC livers than in APAP-treated WT controls (Figure 5A). Consistently, ME1-deficient livers showed higher expression of ER stress-related genes, including PDI, PERK, ATF4, CHOP, ATF6, and sXBP1, following APAP exposure (Figure 5B). Immunoblotting further demonstrated enhanced activation of ER stress-associated signaling, as reflected by increased levels of p-PERK, p-eIF2α, ATF4, GRP78/BiP, and XBP1s in APAP-treated ME1ΔHC livers (Figure 5B). In agreement with these molecular findings, CHOP immunohistochemical staining showed a larger CHOP-positive area in ME1ΔHC livers after APAP challenge (Figure 5C). Collectively, these results indicate that ME1 deficiency does not substantially alter the examined indices of APAP metabolic activation but is associated with aggravated oxidative stress, mitochondrial dysfunction, and ER stress during APAP-induced liver injury.

### 3.4. ME1 Catalytic Activity Supports Redox Buffering in APAP-Treated Hepatocytes

**Figure 6:**
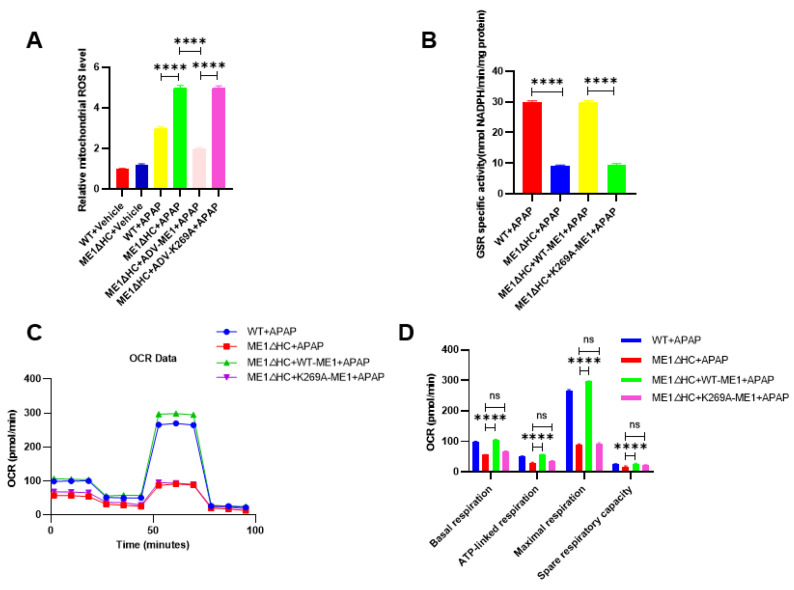
ME1 catalytic activity preserves mitochondrial redox control and respiratory capacity in APAP-treated hepatocytes. Primary hepatocytes from WT and ME1ΔHC mice were treated with APAP as indicated. ME1-deficient hepatocytes were reconstituted with wild-type ME1 or the catalytically inactive K269A-ME1 mutant before APAP challenge. (**A**) Mitochondrial ROS levels were measured in the indicated groups (n = 3 per group). (**B**) Glutathione reductase (GSR) specific activity was determined as a functional readout of NADPH-dependent antioxidant capacity. Enzyme activity was normalized to total protein content and expressed as nmol NADPH/min/mg protein. (n = 3 per group). (**C**) Mitochondrial respiration was assessed in intact primary hepatocytes using Seahorse extracellular flux analysis in assay medium containing glucose, pyruvate, and L-glutamine. Representative oxygen consumption rate (OCR) curves are shown (n = 3 per group). (**D**) Quantification of basal respiration, ATP-linked respiration, maximal respiration, and spare respiratory capacity derived from OCR measurements under mixed substrate availability and normalized to cell number (n = 3 per group). Data are presented as mean ± SD from three independent experiments. Statistical comparisons were performed using one-way ANOVA followed by Tukey’s post hoc multiple comparisons test. ns, not significant; **** *p* < 0.0001, as indicated.

To further strengthen the functional assessment of redox alterations, we examined mitochondrial ROS production and glutathione reductase (GSR) specific activity in APAP-treated primary hepatocytes. Compared with WT hepatocytes, ME1-deficient hepatocytes displayed higher mitochondrial ROS levels after APAP exposure. Reconstitution with wild-type ME1 reduced mitochondrial ROS accumulation, whereas the catalytically inactive K269A-ME1 mutant failed to confer a comparable effect (Figure 6A). Consistently, GSR-specific activity, normalized to total protein content, was markedly reduced in ME1-deficient hepatocytes after APAP challenge and was restored by wild-type ME1 reconstitution, but not by K269A-ME1 (Figure 6B). These results provide functional evidence that ME1 catalytic activity supports NADPH-dependent antioxidant buffering under APAP-induced stress.

### 3.5. ME1 Catalytic Activity Preserves Mitochondrial Respiratory Capacity in APAP-Treated Hepatocytes

We next directly assessed mitochondrial respiratory function using Seahorse extracellular flux analysis in intact primary hepatocytes supplied with glucose, pyruvate, and L-glutamine. ME1-deficient hepatocytes showed impaired oxygen consumption after APAP treatment, as reflected by reduced basal respiration, ATP-linked respiration, maximal respiration, and spare respiratory capacity compared with WT hepatocytes (Figure 6C,D). Reconstitution with wild-type ME1 restored these OCR parameters, whereas K269A-ME1 failed to produce a comparable rescue effect (Figure 6C,D). Therefore, these OCR data reflect integrated mitochondrial respiratory capacity under mixed substrate availability rather than respiration driven specifically by L-malate. Together, these findings indicate that catalytically active ME1 contributes to the preservation of mitochondrial respiratory capacity during APAP-induced hepatocyte stress.

### 3.6. ME1 Overexpression or Reconstitution Restrains ER Stress, Whereas ER Stress Inhibition Partially Rescues ME1-Deficient Injury

**Figure 7:**
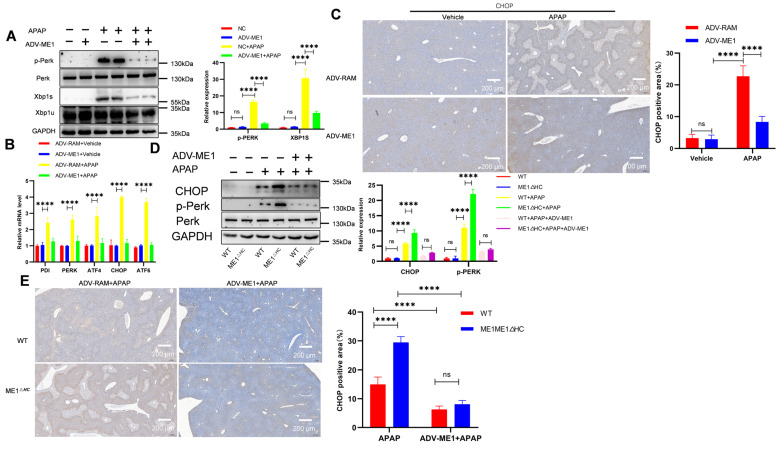
Hepatic overexpression or re-expression of ME1 restrains ER stress signaling in APAP-induced liver injury. Adenoviral delivery of empty vector (ADV-RAM) or ME1 (ADV-ME1) was performed in WT and ME1ΔHC mice 5 days before APAP treatment, as indicated. (**A**) Immunoblot analysis of p-PERK, PERK, XBP1s, and XBP1u in liver tissues from WT mice treated with vehicle or APAP, with or without adenoviral ME1 overexpression, together with quantitative densitometry. (**B**) qRT-PCR analysis of ER stress-related genes (PDI, PERK, ATF4, CHOP, and ATF6) in liver tissues from WT mice of the indicated groups. (**C**) Representative CHOP immunohistochemical staining in liver sections from WT mice treated with ADV-RAM or ADV-ME1 in the presence or absence of APAP, together with quantification of the CHOP-positive area (scale bar: 200 μm). (**D**) Immunoblot analysis of CHOP, p-PERK, and PERK in liver tissues from WT and ME1ΔHC mice after APAP treatment with or without adenoviral ME1 re-expression, together with quantitative densitometry. (**E**) Representative CHOP immunohistochemical staining in liver sections from WT and ME1ΔHC mice following APAP challenge with ADV-RAM or ADV-ME1, together with quantification of the CHOP-positive area (scale bar: 200 μm). Data are presented as mean ± SD. Phosphorylated proteins were normalized to their corresponding total proteins, and non-phosphorylated proteins were normalized to GAPDH. Statistical comparisons were analyzed by one-way ANOVA followed by Tukey’s multiple comparisons test. ns, not significant; **** *p* < 0.0001, as indicated.

To further determine whether ME1 expression is inversely associated with ER stress activation during APAP-induced liver injury, we next examined the effects of hepatic ME1 overexpression and reconstitution. In WT mice, adenoviral ME1 overexpression reduced the APAP-induced elevation of p-PERK and XBP1s, indicating attenuation of ER stress-associated signaling in the liver (Figure 7A). Consistently, ME1 overexpression also suppressed the APAP-triggered increase in the mRNA expression of ER stress-related genes, including PDI, PERK, ATF4, CHOP, and ATF6 (Figure 7B). In agreement with these molecular findings, CHOP immunohistochemical staining showed that ADV-ME1 decreased the hepatic CHOP-positive area in WT mice after APAP administration (Figure 7C).

A similar pattern was observed in the reconstitution experiments. Restoration of ME1 in ME1ΔHC mice reduced hepatic CHOP and p-PERK expression after APAP challenge (Figure 7D). Consistently, CHOP immunohistochemical staining demonstrated that ME1 re-expression decreased hepatic CHOP immunoreactivity in APAP-treated WT and ME1ΔHC mice relative to their corresponding ADV-RAM-treated controls (Figure 7E). Together with the loss-of-function data, these findings support the interpretation that increased ME1 expression is associated with restrained ER stress signaling during APAP-induced liver injury.

**Figure 8:**
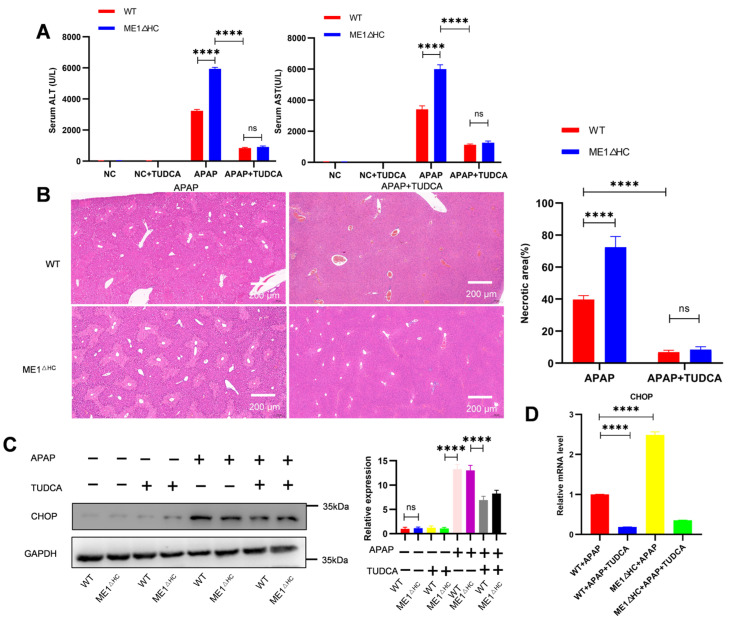
Pharmacological inhibition of ER stress partially rescues the aggravated injury phenotype associated with ME1 deficiency. APAP was administered to WT and ME1ΔHC mice in the presence or absence of TUDCA, as indicated. (**A**) Serum ALT and AST levels were quantified in WT and ME1ΔHC mice from each experimental group (n = 5 mice/group). (**B**) Hepatic histopathological changes were evaluated via H&E staining (scale bar: 200 μm), and the protective effect of TUDCA on APAP-induced hepatic necrosis was assessed by quantification of necrotic areas (n = 5 per group). (**C**) CHOP expression in liver tissues from WT and ME1ΔHC mice under the specified treatments was detected by immunoblot analysis, with corresponding quantitative densitometry (n = 5 per group). (**D**) Quantitative assessment of relative CHOP transcript levels in liver tissues from the specified groups (n = 5 per group). All results are expressed as mean ± SD. Statistical comparisons were performed using one-way ANOVA with Tukey’s post hoc multiple comparisons test. ns, not significant; **** *p* < 0.0001, as indicated.

To further evaluate whether exaggerated ER stress contributes functionally to the aggravated injury phenotype caused by ME1 deficiency, we treated mice with the ER stress inhibitor TUDCA after APAP administration. TUDCA lowered serum ALT and AST levels in APAP-treated mice (Figure 8A), reduced the extent of hepatic necrosis (Figure 8B), and downregulated hepatic CHOP expression (Figure 8C,D). These protective effects were also observed in hepatocyte-specific ME1-deficient mice. Thus, pharmacological attenuation of ER stress partially offsets the detrimental consequences of ME1 deficiency, supporting the view that exaggerated ER stress represents an important downstream component of the ME1-deficient phenotype.

### 3.7. Catalytically Active ME1 Supports NADPH/NADP Balance, AMPK/mTOR-Associated Signaling, and Autophagic Flux in APAP-Treated Hepatocytes

**Figure 9:**
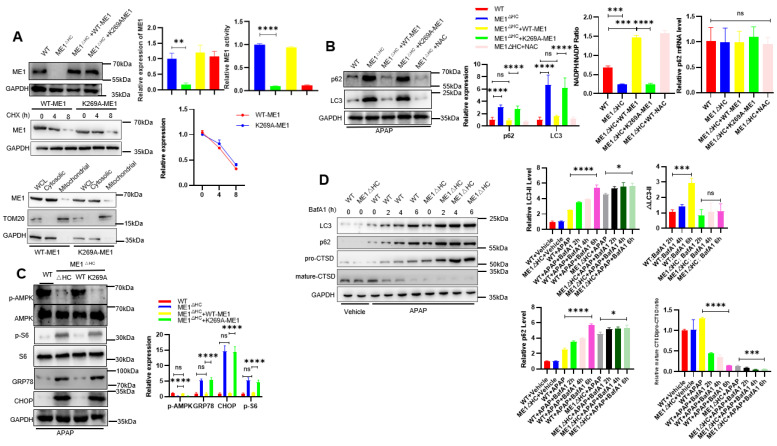
ME1 catalytic activity supports NADPH/NADP balance, AMPK/mTOR-associated signaling, and autophagic flux in APAP-treated hepatocytes. Primary hepatocytes isolated from WT or ME1ΔHC mice were subjected to the indicated treatments. (**A**) ME1-deficient hepatocytes were reconstituted with wild-type ME1 or the catalytically inactive K269A-ME1 mutant. ME1 protein expression was examined by immunoblotting, and ME1 enzymatic activity was measured. Protein stability was assessed by cycloheximide (CHX) chase analysis at 0, 4, and 8 h. Subcellular fractionation was performed to evaluate the distribution of WT-ME1 and K269A-ME1 in whole-cell lysate (WCL), cytosolic, and mitochondrial fractions. GAPDH and TOM20 were used as cytosolic and mitochondrial markers, respectively (n = 3 independent experiments). (**B**) NADPH/NADP ratio, p62 and LC3 protein expression, and p62 mRNA levels were examined in APAP-treated hepatocytes after the indicated treatments. NAC was used to evaluate whether restoration of antioxidant/reductive capacity attenuated autophagy-associated protein accumulation in ME1-deficient hepatocytes (n = 3 independent experiments). (**C**) Immunoblot analysis of p-AMPK, AMPK, p-S6, S6, GRP78/BiP, and CHOP in APAP-treated hepatocytes from the indicated groups, with corresponding densitometric quantification. p-AMPK and p-S6 were normalized to total AMPK and S6, respectively, and non-phosphorylated proteins were normalized to GAPDH. (n = 3 independent experiments). (**D**) Autophagic flux was assessed using a BafA1 time-course assay. APAP-treated WT and ME1ΔHC hepatocytes were exposed to BafA1 for 0, 2, 4, or 6 h before harvesting. LC3, p62, pro-CTSD, and mature CTSD were examined by immunoblotting. Densitometric quantification of LC3-II, ΔLC3-II, p62, and the mature CTSD/pro-CTSD ratio is shown. ΔLC3-II was calculated as the increase in LC3-II abundance after BafA1 treatment relative to APAP-treated cells without BafA1 within the same genotype (n = 3 independent experiments). Data are presented as mean ± SD from three independent experiments. Statistical comparisons were performed using one-way ANOVA followed by Tukey’s post hoc multiple comparisons test. ns, not significant; * *p* < 0.05; ** *p* < 0.01; *** *p* < 0.001; **** *p* < 0.0001, as indicated.

To further define how ME1 influences hepatocyte responses to APAP, we first examined whether its enzymatic activity was required for protection. ME1-deficient primary hepatocytes were reconstituted with either wild-type ME1 or the catalytically inactive K269A mutant. Immunoblotting showed that both wild-type ME1 and K269A-ME1 restored ME1 protein expression in ME1-deficient hepatocytes. However, only wild-type ME1 restored ME1 enzymatic activity, whereas the K269A mutant failed to recover catalytic activity (Figure 9A). These results validated the reconstitution system and allowed us to distinguish the contribution of ME1 catalytic activity from ME1 protein expression alone. We further examined whether the loss of protection by K269A-ME1 could be attributed to altered protein localization or instability. Subcellular fractionation showed that WT-ME1 and K269A-ME1 displayed comparable cytosolic distribution, with minimal mitochondrial enrichment. In addition, cycloheximide chase analysis showed similar degradation kinetics between WT-ME1 and K269A-ME1 (Figure 9A). These results suggest that the impaired protective effect of K269A-ME1 is unlikely to result from altered subcellular localization or reduced protein stability.

We next assessed intracellular NADPH redox status and changes in autophagy-associated proteins under APAP challenge. Compared with WT hepatocytes, ME1-deficient hepatocytes exhibited a markedly reduced NADPH/NADP ratio after APAP exposure. Reconstitution with wild-type ME1 restored the NADPH/NADP ratio, whereas the K269A mutant failed (Figure 9B). Under the same conditions, ME1-deficient hepatocytes showed increased accumulation of p62 and LC3-II. These changes were attenuated by wild-type ME1 reconstitution. However, they were not effectively corrected by K269A-ME1, indicating that ME1 catalytic activity is required for limiting the accumulation of autophagy-associated proteins after APAP treatment (Figure 9B). Treatment with NAC partially reduced p62 and LC3-II accumulation in ME1-deficient hepatocytes, supporting the involvement of redox imbalance in these autophagy-related changes. In contrast, p62 mRNA levels were not significantly different among the indicated groups, suggesting that p62 protein accumulation was unlikely to be primarily driven by transcriptional upregulation (Figure 9B).

Because AMPK/mTOR signaling is closely linked to cellular energy stress and autophagy regulation, we next examined whether this pathway was affected by ME1 catalytic activity. In APAP-treated hepatocytes, ME1 deficiency was associated with reduced p-AMPK/AMPK and increased p-S6/S6, together with elevated ER stress markers GRP78/BiP and CHOP (Figure 9C). Reconstitution with wild-type ME1 restored p-AMPK/AMPK, reduced p-S6/S6, and decreased GRP78/BiP and CHOP expression. By contrast, the K269A mutant failed to reproduce these effects (Figure 9C). These findings indicate that catalytically active ME1 is associated with the preservation of AMPK/mTOR-related signaling and the attenuation of ER stress responses in APAP-treated hepatocytes.

To more rigorously determine whether the accumulation of LC3-II and p62 reflected impaired autophagic flux rather than increased autophagosome formation, we performed a BafA1 time-course assay. In APAP-treated WT hepatocytes, BafA1 induced a time-dependent increase in LC3-II, consistent with ongoing autophagic flux. In contrast, ME1-deficient hepatocytes displayed persistent p62 accumulation and a blunted increase in ΔLC3-II after BafA1 treatment, suggesting impaired autophagic degradation (Figure 9D). To further assess lysosomal involvement, we examined cathepsin D maturation. APAP treatment reduced the mature CTSD/pro-CTSD ratio, with the reduction more pronounced in ME1-deficient hepatocytes (Figure 9D). These findings suggest impaired lysosomal proteolytic maturation or degradation capacity in ME1-deficient hepatocytes after APAP challenge.

Collectively, these data indicate that ME1 catalytic activity contributes to maintaining the NADPH/NADP balance and is associated with preservation of AMPK/mTOR-related signaling, autophagic flux, and ER stress adaptation in APAP-treated hepatocytes. These results support a model in which ME1 deficiency compromises hepatocyte stress adaptation downstream of APAP metabolic activation.

### 3.8. AMPK-Associated Signaling Contributes to ME1-Mediated Regulation of Autophagy and ER Stress

**Figure 10:**
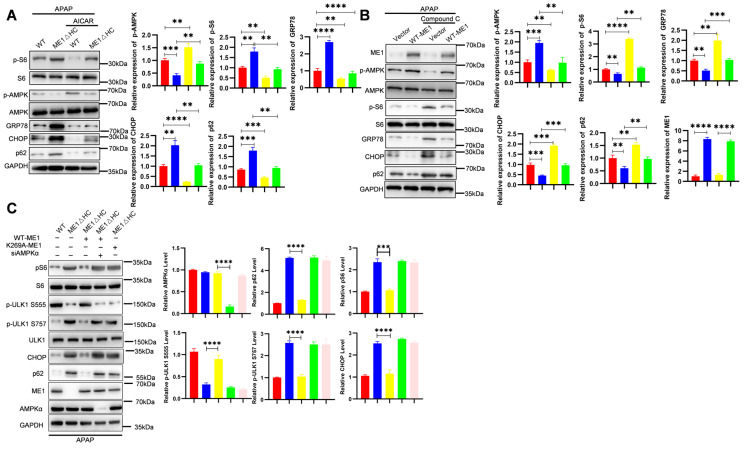
AMPK-associated signaling contributes to the downstream protective effects of catalytically active ME1 in APAP-treated hepatocytes. Primary hepatocytes were subjected to APAP treatment with or without AMPK modulation as indicated. (**A**) WT and ME1ΔHC hepatocytes were treated with APAP in the presence or absence of AICAR. Immunoblot analysis was performed to examine p-S6, S6, p-AMPK, AMPK, GRP78/BiP, CHOP, p62, and GAPDH, with corresponding densitometric quantification (n = 3 independent experiments). (**B**) ME1-deficient hepatocytes were reconstituted with vector or WT-ME1 and treated with APAP in the presence or absence of Compound C. Immunoblot analysis was performed to examine ME1, p-AMPK, AMPK, p-S6, S6, GRP78/BiP, CHOP, p62, and GAPDH, with corresponding densitometric quantification (n = 3 independent experiments). (**C**) ME1-deficient hepatocytes were reconstituted with WT-ME1 or K269A-ME1, with or without AMPKα knockdown, followed by APAP treatment. Immunoblot analysis was performed to examine p-S6, S6, p-ULK1 Ser555, p-ULK1 Ser757, ULK1, CHOP, p62, ME1, AMPKα, and GAPDH, with corresponding densitometric quantification. p-S6, p-ULK1 Ser555, and p-ULK1 Ser757 were normalized to their respective total protein levels. Non-phosphorylated proteins were normalized to GAPDH. (n = 3 independent experiments). Data are presented as mean ± SD from three independent experiments. Statistical comparisons were performed using one-way ANOVA followed by Tukey’s post hoc multiple comparisons test. ns, not significant; ** *p* < 0.01; *** *p* < 0.001; **** *p* < 0.0001, as indicated.

To further examine whether AMPK contributed to the downstream responses associated with ME1 activity, we first used pharmacological modulators of AMPK signaling. In APAP-treated hepatocytes, AICAR increased p-AMPK and reduced p-S6, GRP78/BiP, CHOP, and p62 accumulation in both WT and ME1ΔHC cells (Figure 10A). Conversely, Compound C attenuated the ability of WT-ME1 reconstitution to restore p-AMPK and to suppress p-S6, GRP78/BiP, CHOP, and p62 in ME1-deficient hepatocytes (Figure 10B).

To further strengthen the mechanistic link between ME1 and AMPK-associated signaling, we performed AMPKα knockdown in ME1-reconstituted hepatocytes. Re-expression of WT-ME1, but not the catalytically inactive K269A mutant, reduced p-S6 and p-ULK1 Ser757, increased p-ULK1 Ser555, and attenuated CHOP and p62 accumulation after APAP exposure. Importantly, AMPKα knockdown largely abolished these effects of WT-ME1, leading to increased p-S6, p-ULK1 Ser757, CHOP, and p62, together with reduced p-ULK1 Ser555 (Figure 10C). These findings suggest that catalytically active ME1 restrains APAP-induced stress responses, at least in part, through AMPK-associated autophagy signaling.

### 3.9. Autophagy Modulation Functionally Affects the ME1-Related Injury Phenotype

**Figure 11:**
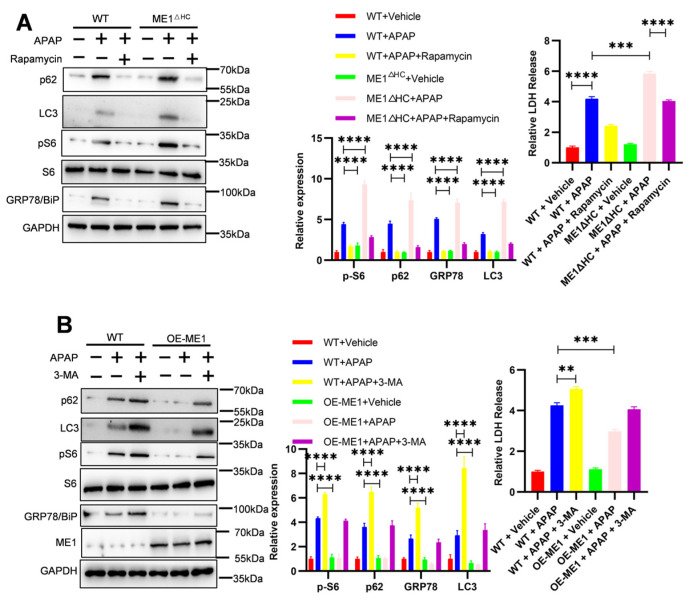
Autophagy modulation functionally affects the ME1-related injury phenotype. As specified in the experimental design, primary hepatocytes were challenged with APAP in the presence or absence of rapamycin or 3-MA. (**A**) Immunoblot analysis of p-S6, S6, p62, LC3, and GRP78/BiP in WT and ME1ΔHC hepatocytes treated with APAP with or without rapamycin, together with quantification of the indicated proteins and LDH release (n = 3 independent experiments). (**B**) Immunoblot analysis was used to detect the expression of p-S6, S6, p62, LC3, GRP78/BiP, and ME1 in hepatocytes with or without ME1 overexpression, and densitometric quantification of target proteins and LDH release was performed (n = 3 independent experiments). p-S6 was normalized to total S6, and non-phosphorylated proteins were normalized to GAPDH. Data are presented as mean ± SD from three independent experiments. Statistical comparisons were performed using one-way ANOVA followed by Tukey’s post hoc multiple comparisons test. ns, not significant; ** *p* < 0.01; *** *p* < 0.001; **** *p* < 0.0001, as indicated.

To assess the functional relevance of autophagy-related changes, we next performed pharmacological intervention experiments. After APAP exposure, rapamycin reduced p-S6, p62, LC3, and GRP78/BiP levels in ME1-null hepatocytes, with a concomitant decrease in LDH release (Figure 11A). Conversely, 3-MA pretreatment markedly weakened the hepatoprotective effect of ME1 overexpression; this was accompanied by higher levels of p-S6, p62, GRP78/BiP, and LC3, along with increased LDH release (Figure 11B). Taken together, these data support a functional contribution of autophagy-related responses to the altered susceptibility of ME1-deficient hepatocytes to APAP-induced injury.

Overall, these findings support a working model in which catalytically active ME1 helps preserve intracellular redox balance and mitochondrial function, thereby supporting AMPK/mTOR-associated autophagy regulation and limiting ER stress in APAP-treated hepatocytes.

### 3.10. Pharmacological Modulation of ME1-Related Pathways Is Associated with Partial Protection Against APAP-Induced Hepatotoxicity

**Figure 12:**
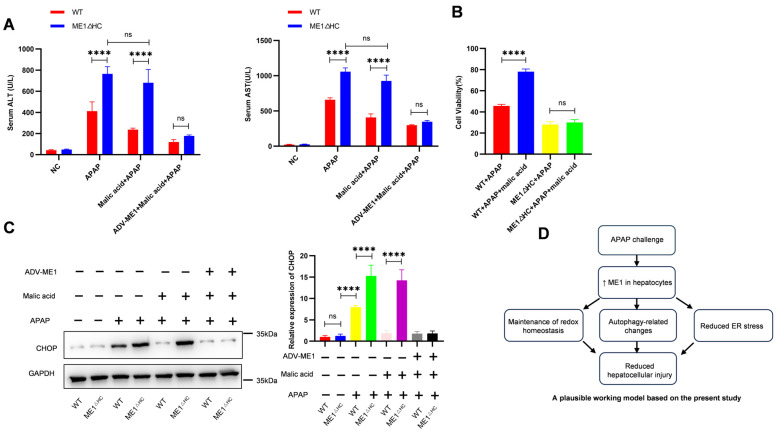
Pharmacological modulation of ME1-related pathways is associated with partial protection against APAP-induced hepatotoxicity. APAP challenge was applied to WT and ME1ΔHC mice or primary hepatocytes, with or without malic acid administration as specified. (**A**) Serum ALT and AST in mice treated with APAP, malic acid, or the indicated combinations, with or without adenoviral ME1 re-expression where applicable (n = 5 per group). (**B**) Viability of APAP-challenged WT and ME1ΔHC primary hepatocytes was measured with or without malic acid supplementation, showing an attenuated but partially preserved protective effect in ME1-deficient hepatocytes (n = 3 independent experiments). (**C**) WT and ME1ΔHC mice were subjected to APAP challenge in the presence or absence of malic acid, as indicated. CHOP expression in liver tissues of each group was detected by immunoblot, with densitometric quantification (n = 5 per group). (**D**) Schematic model depicting the potential role of ME1 in maintaining redox homeostasis, regulating autophagy, and alleviating ER stress during APAP-induced liver injury. Data are presented as mean ± SD. Statistical comparisons were performed using one-way ANOVA followed by Tukey’s post hoc multiple comparisons test. ns, not significant; **** *p* < 0.0001, as indicated.

We further explored whether pharmacological modulation of ME1-related pathways conferred protective effects against APAP-induced liver injury. In vivo, malic acid treatment attenuated APAP-induced injury, as reflected by improved serum liver injury indices (Figure 12A). While malic acid treatment effectively enhanced the survival of APAP-exposed wild-type hepatocytes, this beneficial outcome was significantly diminished in hepatocytes lacking ME1 (Figure 12B). However, the protective effect was not completely abolished in ME1-deficient hepatocytes, indicating that malic acid may exert both ME1-associated and ME1-independent effects. In parallel, lowered CHOP levels were observed in APAP-treated hepatic tissue following malic acid supplementation, and restoration of ME1 expression exerted a similar suppressive effect (Figure 12C). These findings suggest that pharmacological modulation of ME1-related pathways is linked to partial amelioration of APAP-induced liver injury in both in vivo and in vitro contexts. The final schematic diagram summarizes the proposed molecular regulatory framework, in which ME1 contributes to maintaining redox homeostasis, modulating autophagic signaling pathways, and alleviating ER stress in APAP-induced hepatic injury (Figure 12D).

## 4. Discussion

Our findings indicate that hepatocyte-derived ME1 functions as an endogenous protective factor in AILI, and they broaden the current understanding of ME1 beyond its conventional metabolic role. Several findings from this work deserve emphasis. First, the complementary knockout, overexpression, and rescue experiments consistently indicate that ME1 limits APAP-induced hepatic injury in vivo. Second, the aggravated phenotype caused by ME1 deficiency was not accompanied by clear alterations in APAP metabolic activation, suggesting that ME1 acts mainly at the level of downstream stress adaptation rather than toxic metabolite generation itself. Third, the hepatoprotective effect of ME1 was associated with its enzymatic activity and coordinated alterations in redox homeostasis, autophagic flux, and ER stress-associated signaling pathways. Lastly, the findings obtained with malic acid suggest that ME1-related pathways may have potential for pharmacological intervention in AILI.

A key distinction of the current work is that, unlike many previous studies in AILI, it does not focus solely on a single downstream stress pathway. Existing studies have shown that ER stress participates in APAP-induced hepatotoxicity, while enhancement of autophagy via AMPK/mTOR-related signaling is associated with reduced liver injury [18,19]. These studies have been important in defining the relevance of ER stress and autophagy in AILI. However, they did not directly address whether an upstream metabolic regulator could coordinate both processes in the same cellular context. In contrast, our findings support the idea that ME1 may act at the interface of intracellular redox regulation and stress-response pathways. This distinction matters because APAP hepatotoxicity begins with metabolic injury but progresses through a wider pathogenic network involving oxidant stress, mitochondrial dysfunction, organelle damage, and maladaptive stress signaling [21,22,23].

The pathophysiological significance of our findings is likely rooted in the role of ME1 in maintaining intracellular reductive capacity. Because ME1 produces NADPH in the cytosol, it is in a favorable position to contribute to glutathione-dependent antioxidant defense and other adaptive cellular programs [1]. In our experimental models, loss of ME1 was accompanied by enhanced lipid peroxidation and more pronounced energetic disturbance. In contrast, the K269A mutant, which lacks catalytic activity, did not reproduce the downstream phenotype seen with wild-type ME1. These data are therefore consistent with the interpretation that catalytically active ME1 contributes to hepatocytes’ ability to buffer APAP-induced stress. The functional redox and mitochondrial assays added in the revised study further strengthen this interpretation. ME1-deficient hepatocytes displayed increased mitochondrial ROS, reduced GSR activity, and impaired mitochondrial respiration after APAP challenge, whereas reconstitution with WT-ME1, but not K269A-ME1, restored these parameters. These findings suggest that ME1 catalytic activity contributes functionally to antioxidant capacity and mitochondrial respiratory adaptation rather than merely correlating with redox marker changes. The relationship between ME1 activity and the NADP(H)- and glutathione-dependent redox systems should be interpreted in this biochemical context. ME1 directly generates NADPH from NADP+ during malate decarboxylation; therefore, loss of ME1 catalytic activity would be expected to reduce the cytosolic NADPH/NADP+ ratio under conditions in which compensatory NADPH-producing pathways are insufficient. NADPH, in turn, provides reducing equivalents for glutathione reductase, which regenerates GSH from GSSG. Thus, the reduced NADPH/NADP+ ratio and GSR specific activity, together with increased mitochondrial ROS and lipid peroxidation, support the interpretation that ME1 deficiency weakens NADPH-dependent antioxidant buffering during APAP stress. However, because the GSH/GSSG ratio did not show a large genotype-dependent difference in all comparisons and formal redox potentials were not calculated using the Nernst equation, our data should be interpreted as evidence of impaired redox buffering capacity rather than a complete quantitative definition of cellular redox potential. We do not interpret these findings to mean that ME1 is the predominant NADPH-generating pathway in hepatocytes. Other systems, including the pentose phosphate pathway and NADP-dependent isocitrate dehydrogenases, likely provide substantial NADPH under basal and stress conditions. The present data instead suggest that ME1 supplies a functionally relevant, cytosolic NADPH-linked contribution that becomes important for stress adaptation during APAP-induced injury. Importantly, the present results do not indicate that ME1 prevents the initial generation of the toxic APAP metabolite. Rather, they suggest that ME1 influences how hepatocytes cope with the injury that follows bioactivation. This interpretation is aligned with the current understanding that redox imbalance, mitochondrial dysfunction, and energetic failure are central components of AILI pathogenesis after the initiating metabolic event [21,24,25].

Another important aspect of the present work is the relationship between ME1, autophagic flux, and ER stress. In ME1-deficient hepatocytes, the increase in p62 and altered LC3-II dynamics, when considered together with the BafA1 time-course data, are better interpreted as evidence of impaired autophagic flux rather than increased autophagosome formation. This interpretation is further supported by the reduced mature CTSD/pro-CTSD ratio, which suggests impaired lysosomal proteolytic maturation or degradation capacity in ME1-deficient hepatocytes after APAP challenge. Therefore, ME1 deficiency appears to compromise autophagic degradation, accompanied at least in part by impaired lysosome-associated proteolytic maturation or degradation capacity. Nevertheless, because autophagy is a dynamic, multi-step process, additional flux-specific approaches, such as tandem fluorescent LC3 reporter assays, would further refine the spatial and temporal interpretation of these changes [26].

The reduced ATP/ADP ratio, impaired mitochondrial respiration, and increased mitochondrial ROS observed in ME1-deficient hepatocytes provide a plausible metabolic context for altered AMPK/mTOR-autophagy signaling. The siAMPKα experiment further supports the involvement of AMPK-associated signaling in the downstream protective pattern induced by catalytically active ME1. However, these findings do not establish whether AMPK activation is a primary driver of ME1-mediated protection or a secondary adaptive response to improved redox and bioenergetic status.

Pharmacological modulation using AICAR, Compound C, rapamycin, and 3-MA further supports the involvement of AMPK/mTOR-associated autophagy regulation in ME1-related hepatocyte stress adaptation [10,19]. However, these findings should be interpreted with caution. Our data support a working model in which catalytically active ME1 is associated with preserved autophagic flux and reduced ER stress. However, they do not allow a definitive linear order to be assigned to redox disturbance, AMPK/mTOR signaling, autophagy, and ER stress. Because these pathways are highly interconnected in injured hepatocytes, it is more likely that ME1 participates in a broader adaptive network than in a simple one-way signaling axis [18].

From a translational perspective, the malic acid experiments are encouraging but should be interpreted conservatively. Partial attenuation of APAP-induced hepatotoxicity by malic acid was observed in both animal and cell-based experiments, although this effect appeared weaker in the absence of ME1. Importantly, the protective effect of malic acid was attenuated but not completely abolished in ME1-deficient settings, suggesting that malic acid is not a selective ME1-directed intervention and may also act through ME1-independent metabolic or mitochondrial mechanisms. In addition, the doses of malic acid and TUDCA used in this study were selected based on experimental efficacy rather than formal dose–response or safety analyses in healthy animals. We also did not directly determine whether malic acid increased hepatic ME1 activity or NADPH levels in vivo. Therefore, the present pharmacological findings should be interpreted as preliminary pathway-modulation data rather than evidence for a selective ME1-targeted therapy, and these issues should be addressed in future pharmacokinetic and pharmacodynamic studies [5,6].

The present study also has several limitations that merit consideration. One limitation is that only male mice were used in the present study. Given the known sex differences in APAP metabolism, CYP450 expression, and susceptibility to APAP-induced liver injury, future studies should determine whether the protective effect of ME1 is conserved in female mice. We extended our analysis of oxidative damage by examining GSSG, the GSH/GSSG ratio, and MDA, whereas additional indices of oxidative and lipid injury were not determined. A broader redox profiling strategy would help refine the interpretation of ME1-dependent stress adaptation. The present work supports the involvement of AMPK/mTOR-associated signaling and autophagic flux, but does not fully resolve the temporal order or relative causal weight of these pathways. Although the malic acid data are suggestive, additional studies will be required to define pharmacokinetics, dose–response relationships, therapeutic timing, and safety. Validation in human hepatocytes or human liver organoid models will also be needed before the translational relevance of ME1-related modulation can be established. Finally, because ME1 is embedded in wider metabolic networks, it will be important to determine whether its protective effect intersects with other adaptive programs relevant to APAP injury, including mitochondrial quality control, ferroptosis-related processes, and nuclear metabolic regulators. Genetic or flux-specific tools would further strengthen the interpretation of autophagy-related changes beyond pharmacological intervention [22,23]. Although APAP-protein adduct analysis was added in the revised study to strengthen the assessment of APAP metabolic activation, direct CYP enzymatic activity was not measured. Because Seahorse OCR was measured in intact hepatocytes supplied with glucose, pyruvate, and L-glutamine, these data reflect integrated mitochondrial respiratory capacity under mixed substrate conditions. L-malate-specific respiration was not directly assessed in permeabilized hepatocytes or isolated mitochondria, which remains a limitation of the present study. In addition, although the BafA1 time-course assay and CTSD maturation analysis improved the interpretation of autophagic flux and lysosomal degradation, tandem fluorescent LC3 reporter assays would provide additional spatial and temporal information. Finally, GSR activity was measured as a functional readout of NADPH-dependent antioxidant capacity, whereas other NADPH-dependent systems, such as thioredoxin reductase, remain to be examined in future studies. Our results support the view that hepatocyte-derived ME1 helps protect against APAP-induced liver injury. ME1 appears to help maintain redox balance and to modulate stress responses related to autophagic flux and ER stress. Its effect is more likely to occur downstream of APAP metabolic activation than at the level of toxic metabolite formation. Taken together, these data provide further rationale for investigating ME1-related pathways in AILI.

## 5. Conclusions

Our findings support the view that ME1 exerts a potential protective effect in APAP-induced liver injury. ME1 expression increased after APAP exposure, and the opposing phenotypes observed after ME1 loss or restoration were consistent with this effect. The protective effect mediated by ME1 was unlikely to stem from changes in APAP metabolic activation; instead, it was more closely linked to catalytic activity, the maintenance of redox balance, intact autophagic flux, and reduced ER stress. While malic acid demonstrated partial protective effects in our experimental models, its therapeutic significance remains preliminary. Taken collectively, these results provide further justification for exploring ME1-associated pathways in AILI.

## Figures and Tables

**Table 1:** Antibodies used in this study.

Antibody	Cat No.	Manufacturer	Concentration
ME1	16619-1-AP	proteintech	WB: 1:1000 IHC: 1:200
CYP2E1	19937-1-AP	proteintech	WB: 1:1000
ATF4	11815 S	CST	WB: 1:1000
LC3	14600-1-AP	proteintech	WB: 1:1000
CHOP	15204-1-AP	proteintech	WB: 1:1000 IHC: 1:200
p-eIF2α	3398 S	CST	WB: 1:1000
eIF2α	5324 S	CST	WB: 1:1000
sXBP1	12782 S	CST	WB: 1:1000
tXBP1	ab37152	Abcam	WB: 1:1000
GRP78	11587-1-AP	proteintech	WB: 1:1000
p-PERK	3179 S	CST	WB: 1:1000
PERK	3192 S	CST	WB: 1:1000
GAPDH	ES-Pab001/100	ESScience	WB: 1:3000
p-S6	80206-1-RR	proteintech	WB: 1:1000
S6	80208-1-RR	proteintech	WB: 1:1000
p62	18420-1-AP	proteintech	WB: 1:1000
CTSD	55021-1-AP	proteintech	WB: 1:1000
p-ULK1 Ser555	5869	CST	WB: 1:1000
p-ULK1 Ser757	6888	CST	WB: 1:1000
ULK1	8054	CST	WB: 1:1000
Acetaminophen/APAP-protein adducts	0016-0104	Bio-Rad	WB: 1:1000

**Table 2:** qRT-PCR primer sequences.

Gene	Forward Primer (5′-3′)	Reverse Primer (5′-3′)
*PDI*	CAAGATCAAGCCCCACCTGAT	AGTTCGCCCCAACCAGTACTT
*PERK*	CCGATGTCAGTGACAACAGCTG	AAGACAACGCCAAAGCCACCAC
*ATF4*	AACCTCATGGGTTCTCCAGCGA	CTCCAACATCCAATCTGTCCCG
*CHOP*	GGAGGTCCTGTCCTCAGATGAA	GCTCCTCTGTCAGCCAAGCTAG
*ATF6*	GTCCAAAGCGAAGAGCTGTCTG	AGAGATGCCTCCTCTGATTGGC
*S-XBP1*	GGTCTGCTGAGTCCGCAGCAGG	GGGGAAGGACATTTGAAACA
*GAPDH*	CATCACTGCCACCCAGAAGACTG	ATGCCAGTGAGCTTCCCGTTCAG
*P62*	GCTCTTCGGAAGTCAGCAAACC	GCAGTTTCCCGACTCCATCTGT

## Data Availability

The data presented in this study are available on request from the corresponding author.

## Supplementary Materials

The following supporting information can be downloaded at https://www.mdpi.com/article/10.3390/metabo16060423/s1. Figure S1: Tissue specificity of hepatocyte-specific ME1 deletion. Immunoblot analysis of ME1 expression in extrahepatic tissues, including lung, kidney, and heart, from WT and ME1ΔHC mice. GAPDH was used as the loading control (n = 6 per group). Table S1: Exact *p*-values for the main statistical comparisons. Exact *p*-values are provided for all comparisons where *p* ≥ 0.001. For highly significant comparisons with *p* < 0.0001, threshold notation is retained.

## Abbreviations

CYP2E1	Cytochrome P450 2E1
NAPQI	N-acetyl-p-benzoquinone imine
GSH	Glutathione
UPR	Unfolded protein response
IHC	Immunohistochemistry
TUDCA	Tauroursodeoxycholic acid
ALT	Alanine aminotransferase
AST	Aspartate aminotransferase
qRT-PCR	Quantitative real-time polymerase chain reaction
H&E	Hematoxylin and Eosin
IF	Immunofluorescence
WT	Wild type
APAP	Acetaminophen
AILI	APAP-induced liver injury
ME1	Malic enzyme 1
ADV	Adenovirus
ER	Endoplasmic reticulum
BiP	Binding immunoglobulin protein, also known as GRP78
ATF4	Activating transcription factor 4
PDI	Protein disulfide isomerase
XBP-1	X-box binding protein 1 (XBP1)
CHOP	C/EBP-homologous protein
eIF2α	Eukaryotic initiation factor 2α
PERK	Protein kinase R-like ER kinase
CCK-8	Cell counting kit-8
NAC	N-acetylcysteine
