# Supplementary figures and images for: Malic Enzyme 1 Limits Acetaminophen-Induced Liver Injury by Sustaining Redox and Bioenergetic Homeostasis
Source: Metabolites. 2026 Jun 16;16(6):423. doi: 10.3390/metabo16060423 (PMC13303971; doi:10.3390/metabo16060423)

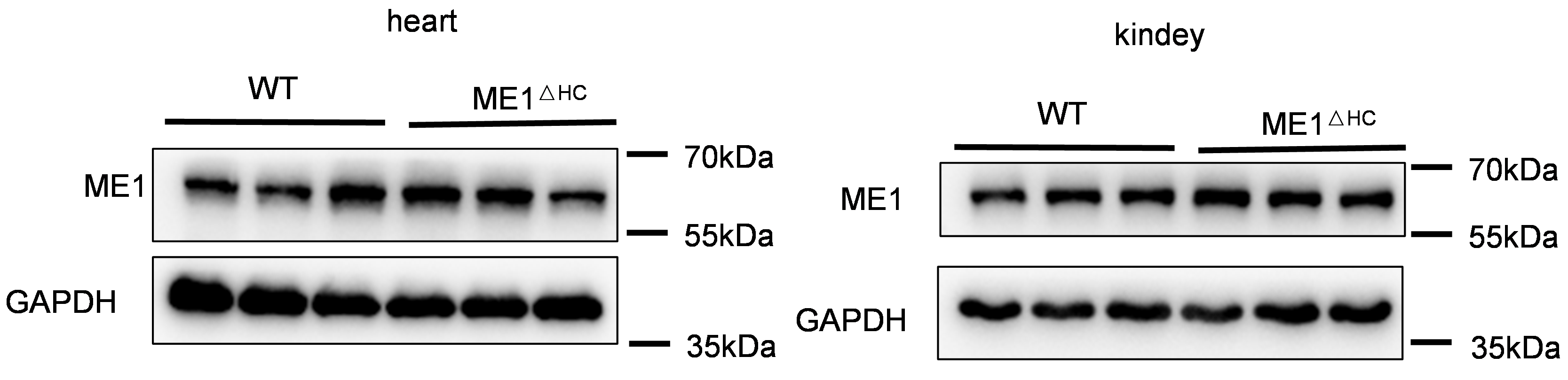

Supplement: Supplementary file 1 [file metabolites-16-00423-s001.zip › Figure S1.tif]
